# Go with the flow—biology and genetics of the lactation cycle

**DOI:** 10.3389/fgene.2015.00118

**Published:** 2015-03-26

**Authors:** Eva M. Strucken, Yan C. S. M. Laurenson, Gudrun A. Brockmann

**Affiliations:** ^1^Animal Science, School of Environmental and Rural Science, University of New EnglandArmidale, NSW, Australia; ^2^Breeding Biology and Molecular Genetics, Faculty of Life Sciences, Humboldt-Universität zu BerlinBerlin, Germany

**Keywords:** time-dependent, longitudinal, lactation curve, breeding value, genome-wide association, genomic selection, genomic prediction

## Abstract

Lactation is a dynamic process, which evolved to meet dietary demands of growing offspring. At the same time, the mother's metabolism changes to meet the high requirements of nutrient supply to the offspring. Through strong artificial selection, the strain of milk production on dairy cows is often associated with impaired health and fertility. This led to the incorporation of functional traits into breeding aims to counteract this negative association. Potentially, distributing the total quantity of milk per lactation cycle more equally over time could reduce the peak of physiological strain and improve health and fertility. During lactation many factors affect the production of milk: food intake; digestion, absorption, and transportation of nutrients; blood glucose levels; activity of cells in the mammary gland, liver, and adipose tissue; synthesis of proteins and fat in the secretory cells; and the metabolic and regulatory pathways that provide fatty acids, amino acids, and carbohydrates. Whilst the endocrine regulation and physiology of the dynamic process of milk production seems to be understood, the genetics that underlie these dynamics are still to be uncovered. Modeling of longitudinal traits and estimating the change in additive genetic variation over time has shown that the genetic contribution to the expression of a trait depends on the considered time-point. Such time-dependent studies could contribute to the discovery of missing heritability. Only very few studies have estimated exact gene and marker effects at different time-points during lactation. The most prominent gene affecting milk yield and milk fat, *DGAT1*, exhibits its main effects after peak production, whilst the *casein* genes have larger effects in early lactation. Understanding the physiological dynamics and elucidating the time-dependent genetic effects behind dynamically expressed traits will contribute to selection decisions to further improve productive and healthy breeding populations.

## Introduction

Lactation is an orchestrated process aimed at providing nutrition and immune protection to the offspring; however, the mother must also retain sufficient resources to ensure her own survival. Thus, the quantity and composition of milk produced is strongly dependent on the developmental stage of the offspring and the maintenance requirements of the mother. As such, milk production is a classic exemplar of a time-dependent dynamic process.

The domestication of animals inevitably led to selective breeding for increased productivity. The uninterrupted increasing global demand for dairy products necessitated a concurrent increase in milk production. Thus, in order to meet market requirements, the dairy sector implemented selective breeding programs which have led to a doubling in the amount of milk produced per cow over the last 50 years, such that total milk production is increasing despite a decline in dairy cattle populations (Food and Agriculture Organization of the United Nations, FAO, 2012^1^). Recently, this has included the implementation advanced breeding programs and the development of tools to utilize genetic and genomic information (Goddard and Hayes, 2007; Seefried et al., 2010). However, increasing the milk production per cow has detrimental effects on animal health and fertility (Ingvartsen et al., 2003; Oltenacu and Broom, 2010). Consequently, breeding goals were adjusted to incorporate health and fertility traits into breeding indices (Osteras et al., 2007; Boichard and Brochard, 2012).

These breeding indices have enabled dairy farmers to breed for milk production and functional traits without requiring knowledge on how these practices impact upon the dynamics of milk production or change the expression of underlying genes. However, the continuous development of genetic and genomic tools, as well as computational capacities, will allow breeders of the future to base their decisions not only on phenotypically observable traits or indirect genetic marker information but also on the direct causative genetic variants.

As with many other complex traits important in livestock production, milk production is influenced by many genetic loci that act directly, interact with each other and/or interact with the environment (Lemay et al., 2009). This makes the study of quantitative traits challenging, especially when time-dependent components are considered. This review details the most important regulators of milk production and their underlying genes in the context of the dynamics of a lactation cycle, and summarizes the efforts made to identify genetic loci affecting the dynamics of milk production during lactation.

## Conflict between production and functional traits

**Figure 1:**
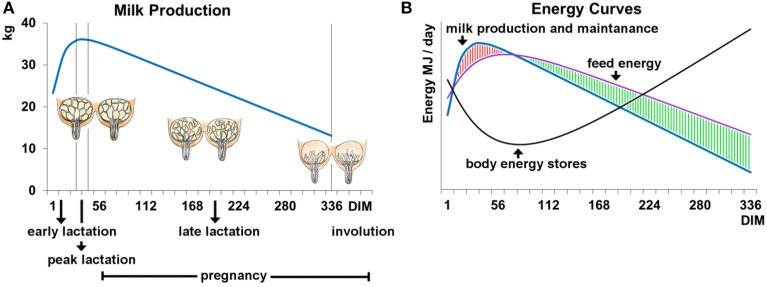
**(A)** Milk production and **(B)** energy supply and requirements during the lactation cycle of 340 days. **(A)** The curve represents the milk yield per day of lactation and reaches a peak production around lactation days 40–50. Shortly before lactation and until peak production the udder and the alveolar system are highly developed. In later lactation the alveolar system regresses continuously until the end of lactation and into involution. **(B)** The blue curve represents the energy that is needed for milk production and maintenance of vital body functions. The energy needed for milk production is highest when milk production reaches a peak. At the same time the energy taken in through food (purple curve) cannot cover the energy requirements for milk production which leads to a loss in body energy stores (black curve). This imbalance in energy homeostasis changes with the decline of milk production in late lactation.

The milk production of a cow follows a dynamic curve (Figure 1A; Stanton et al., 1992). After an initial rapid increase in milk yield during early lactation, milk yield (as well as protein and fat content) peak around 6 weeks into lactation, after which production slowly decreases until the end of lactation. Dairy cows experience an energy deficiency during early and peak lactation (Figure 1B; Collard et al., 2000) due to the high energy requirements for milk production not being met because of physiological limitations which constrain food intake (i.e., bulk capacity; Allen, 1996) and mobilization of bodily energy resources. This energy deficit has been proposed to have detrimental effects on health and fertility which have been reviewed and discussed by Oltenacu and Broom (2010), and negative genetic correlations have been reported between milk production and a variety of functional traits (Zimmermann and Sommer, 1973; Dekkers et al., 1998; Ingvartsen et al., 2003; Muir et al., 2004). However, it has to be noted that total milk yield and the energy balance during early lactation seem to be independent, as correlations have been reported to be very low (Spurlock et al., 2012). Further, the negative impact of lactation on fertility may serve a functional purpose to provide optimal birth spacing for the survival of offspring. Therefore, there may be other endogenous factors yet to be discovered that negatively affect health and fertility traits.

From a nutritionist's point of view it might be necessary to reduce, rather than to increase, peak milk yield in order to decrease the energy deficiency experienced during early and peak lactation, and thereby improve health and fertility traits. However, this is in direct conflict with the desire to increase overall milk production. Therefore, an alternative method of increasing overall milk production might be via increasing production persistency. A better production persistency raises the overall gain per lactation due to an increased persistency affecting the longest part of the lactation (i.e., late lactation; Dekkers et al., 1998; Inchaisri et al., 2011). However, there are some reports indicating that a high persistency may also be antagonistic to the animal's health, and thus also needs to be considered in regards to finding an optimal persistency and lactation duration (Harder et al., 2006; Appuhamy et al., 2009).

Production persistency is most often defined as a lesser decrease in milk production after the peak, i.e., a flatter shape of the lactation curve compared to another animal or the heard average. Such calculations can be based on the difference of peak yield to a 305d measurement, on test-day deviations, or on parameters of lactation curve models (Gengler, 1996). As persistency is negatively correlated to yields, some studies prefer to calculate persistency as a linear regression of test-day deviations on days in milk to achieve a yield independent estimate (Cole and VanRaden, 2006; Cole and Null, 2009). By employing such an estimate, it would enable a breeder to select on milk yield and persistency independently; however, currently only very few breeding companies provide such estimates to their clients.

One problem with persistent production is the requirement to dry-off a cow between lactations. However, if the production system does not require yearly calving, the duration of the lactations can be chosen according to daily yield. Subsequently, with increased lactation duration, the time point of insemination has to be postponed. Assuming that the peak production remains around 6 weeks into the lactation cycle, a later time point for insemination has the added benefit that a new pregnancy begins after the energy deficit caused by the high peak production. Thus, fertility issues potentially arising from an energy deficit will be reduced. Regardless of lactation duration, the general recommendation for days dry is still 45–70 days (Kuhn et al., 2006, 2007; Sawa et al., 2012). The potential implications of increased lactation duration on generation intervals and fewer replacement animals could be counteracted through the utilization of sexed semen to increase the ratio of female calves.

Current methods in animal breeding apply an index of traits weighted according to their economic importance as well as heritability in the breeding goal. Further, phenotypic and genetic correlations between traits within the index are included, on the one hand to increase accuracy on lowly heritable traits, and on the other hand to account for potential negative correlations (Dekkers, 2007).Whilst milk production is still the most important trait in most countries, conformation, udder health, and fertility have been added to balance the negative correlation between a high production and the animal's welfare and longevity (VanRaden, 2004; Miglior et al., 2005). However, the exact impact of such breeding indices on the shape of the lactation curve or the dynamic gene effects remains unknown. In the following section, we look at the physiological interplay that forms the lactation cycle as this is the basis of understanding which genetic factors are ultimately involved.

## Physiology of a dynamic milk production

### Mammogenesis

The development of the mammary gland is the primary factor affecting milk production. A well-developed mammary gland with many fully differentiated secretory cells, good blood supply, and strong connective tissue will be highly productive over a long time.

**Figure 2:**
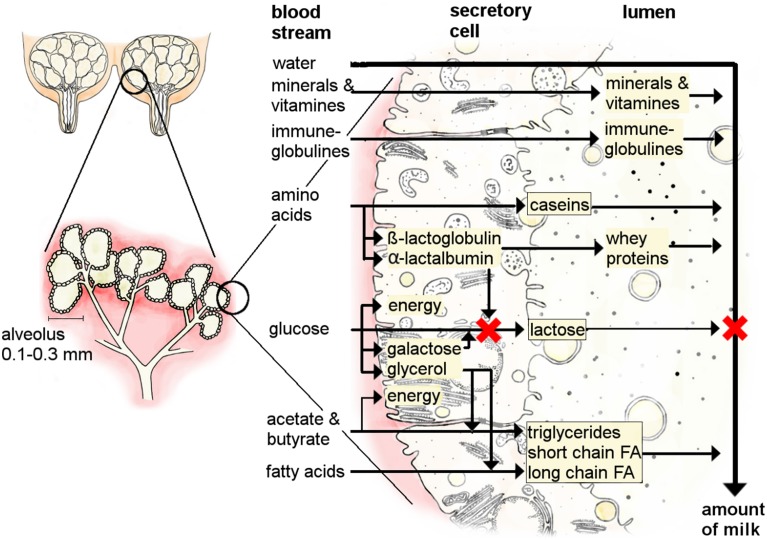
**The process of milk secretion in the udder of a cow (partially adapted from Wattiaux, 1996)**. Milk is secreted in the alveoli system of the mammary gland. Several substances can pass the cell membrane from the blood stream (water, minerals, vitamins, immune-globulins), whilst others need transporters and are produced in the secretory cells (proteins, fat, lactose).

The mammary gland forms a rudimentary duct tree during fetal development in response to maternal hormones (Watson and Khaled, 2008). From birth until puberty, mammary gland growth is due to the formation of a fat-pad rather than the development of specialized mammary gland tissue (McNally and Martin, 2011). At puberty the initiation of the estrus cycle, via follicle-stimulating hormones and luteinizing hormone, stimulates the ovaries to synthesize and release estrogen and progesterone. The concurrent elevations in both estrogen and progesterone orchestrate the main growth of the mammary gland during pregnancy by ductal growth and lobular formation which leads to the formation of lobule-alveoli (Hennighausen and Robinson, 2005; Bloise et al., 2010; Koos, 2011). Alveoli are an accumulation of secretory cells grouped around a hollow center, the lumen, where the milk is stored (Figure 2). Thus, as pregnancy progresses, the adipose cells of the mammary gland are gradually replaced by specialized mammary gland tissue. Mammary gland growth continues during early lactation until peak lactation, after which the mammary gland shrinks due to the rate of secretory cell loss exceeding the rate of cell division (Figure 1A; Capuco and Akers, 1999).

Hormones and growth factors are important in determining how many secretory cells develop, and thus how much milk can be produced in the mammary gland (Watson and Khaled, 2008; McNally and Martin, 2011). By slowing down the process of hormonal stimulation of secretory cell proliferation during late pregnancy and early lactation, and favoring an extended time during which new cells are produced, the peak milk production could be reduced and a better persistency achieved. This may also be achieved by slowing down the rate of cell death which is also regulated through a cascade of hormones and growth factors (Sureshbabu et al., 2011; Watson et al., 2011).

### Milk secretion

**Figure 3:**
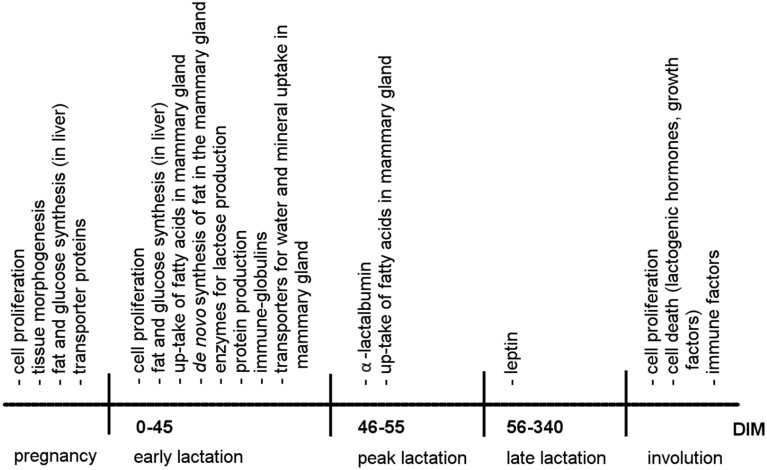
**Chronology of gene expression and physiological processes during a lactation cycle**. DIM, days in milk.

A second crucial point for milk production concerns the quantity and quality of the secreted milk. Milk is an emulsion of fat and water containing dissolved carbohydrates, proteins, vitamins, and minerals that all have to be produced in or transported to the mammary gland. During lactation, quantitative milk yield is primarily regulated by lactose within the alveoli. Alveolar lactose influences the osmotic pressure between blood and alveoli and thereby the amount of water drawn into the alveoli (Figure 2; Zhao and Keating, 2007). Some of the substances in milk such as minerals, vitamins, or immune-globulins pass the cell membranes directly from the blood into the lumen via transporter proteins (Figure 2; Neville and Watters, 1983). The activity of these transporter proteins is increased when milk production starts to enhance the uptake of water into the secretory cells of the mammary gland (Figure 3; Zhao and Keating, 2007; Anantamongkol et al., 2010; Wickramasinghe et al., 2012). Substances such as lactose, proteins and fat have to be synthesized in the secretory cells from components such as glucose, amino acids, triglycerides, or fatty acids that stem from the dietary nutrients or body resources such as adipose tissues or skeletal muscles (Figures 2, 3; Burgoyne and Duncan, 1998; Zhao and Keating, 2007; Bionaz and Loor, 2008b). Lactose is synthesized from blood glucose and galactose (synthesized from glucose) by a lactose synthase enzyme composed of galactosyltransferase and α-lactalbumin in the golgi complex of mammary secretory cells (Figure 2). The amount of glucose in the blood is regulated by energy intake, insulin and leptin (Figure 3; Li et al., 2010).

Proteins and fat are important for qualitative milk yield in terms of organoleptic properties of the milk and downstream industries such as cheese and butter production (Bailey et al., 2005; Bauman et al., 2006). Caseins, α-lactalbumin and β-lactoglobulin represent the main fraction of milk proteins. They are synthesized mainly from amino acids broken down from digested food and transported through the blood stream to the secretory cells (Burgoyne and Duncan, 1998). Milk fat is composed of triglycerides, long- and short-chain fatty acids which are partly synthesized in the liver or in secretory cells of the mammary gland from short-chain dietary lipids that are obtained from the rumen, and partly from mobilized fats from bodily fat depots (Figure 2; Bionaz and Loor, 2008b).

Lactation is coupled with changes in the activity of genes in the mammary gland but also in other organs. In the liver, fat and glucose synthesis is highly increased from pregnancy to early lactation to provide fatty acids and blood glucose for milk production (Figure 3; Bell and Bauman, 1997; Casey et al., 2009), whereas fat synthesis is decreased in adipose tissue and the expression of transporter genes for the uptake of blood glucose into somatic cells is reduced to ensure that nutrients are available for milk production (Bell and Bauman, 1997; Casey et al., 2009).

In conclusion, to understand the genetics behind a lactation cycle, a number of gene pathways need to be considered. These include genes regulating food intake and blood glucose levels; the digestion, absorption, and transportation of nutrients; the activity of the secretory cells in the mammary gland, liver, and adipose tissue; the synthesis of proteins and fat in the secretory cells; and the pathways which provide triglycerides, fatty and amino acids, transporter proteins, and transcription factors.

### Genetics of milk production

The establishment of the *Bos taurus* genome assembly (Bovine HapMap et al., 2009), along with proteome and gene expression studies, have made it possible to estimate the number of genes involved in milk production, from mammogenesis to milk secretion. Between 6000 and 19,000 genes distributed across all 29 bovine autosomes and the X-chromosome have been reported to be differentially expressed during the lactation cycle, though not exclusively in the mammary gland (Lemay et al., 2009; Wickramasinghe et al., 2012). Thus, the genes predicted to be involved (directly or indirectly) in the regulation of milk production, account for between 25 and 75% of all predicted cattle genes (*Bos taurus* UMD 3.1-Primary Assembly, Zimin et al., 2009). Most genes contribute to pathways that directly affect economically important traits such as milk yield and composition. A multitude of genome-wide association studies (GWAS) using high density SNP chip data have previously been conducted to narrow down regions and identify causative genes that affect milk production traits (Cole et al., 2011; Strucken et al., 2012a; Buitenhuis et al., 2014; Raven et al., 2014). Whilst regions and potential genes with effects on milk production traits have been reported for almost all bovine chromosomes, repeatedly occurring genes are located on chromosomes 27, 6, 20, and 14 (Lemay et al., 2009).

**Figure 4:**
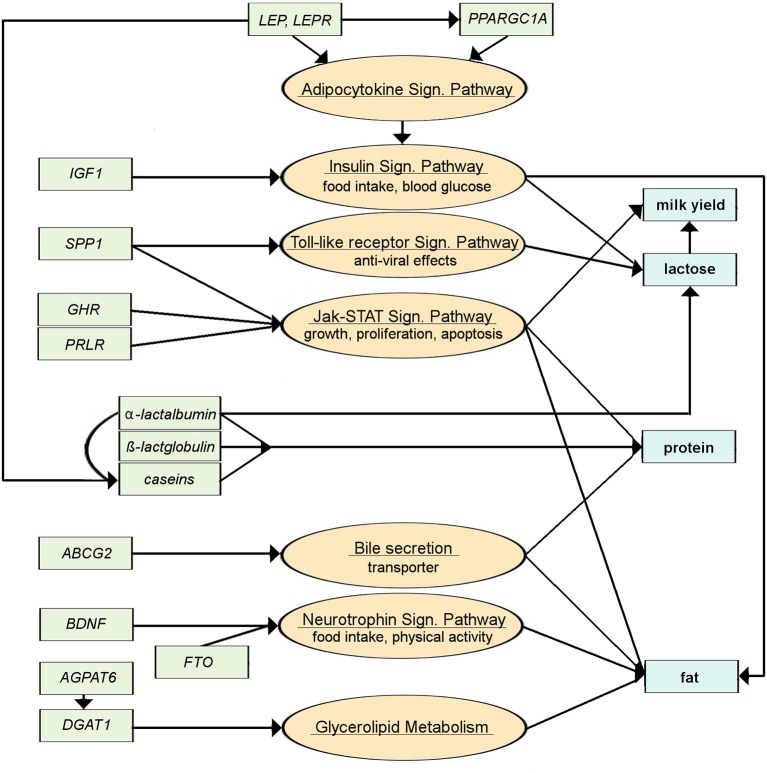
**Simplified pathways for major genes involved in milk production**. Green boxes are genes, orange circles are the pathways the genes are involved in, blue boxes are the milk production traits that are affected (information is assembled from KEGG Pathway Database, 17.11.2014; http://www.genome.jp/kegg/pathway.html and literature review; for gene names, see Table 1).

Only around a dozen candidate genes have been consistently identified between studies and described more extensively with regards to their association with the main milk production traits (Table 1). The pathways through which these genes affect milk production traits depict the variety of processes that have to be considered (Figure 4). Genes like the *BDNF*, *FTO*, or *IGF1* impact upon food intake and thus nutrient and energy availability (Mullen et al., 2011; Zielke et al., 2011, 2013; Waters et al., 2012). Other genes such as *GHR*, *PRLR*, or *SPP1* affect growth, proliferation, and apoptosis of cells (Viitala et al., 2006; Khatib et al., 2007; Banos et al., 2008; Lu et al., 2011a; Rahmatalla et al., 2011), whilst *DGAT1* and *AGPAT6* are involved directly in triglyceride synthesis (Winter et al., 2002; Bionaz and Loor, 2008a; Strucken et al., 2010a; He et al., 2011). Of further note are the *casein* genes which encode the major fraction of milk proteins (Velmala et al., 1995). Figure 4 provides an overview of those candidate genes and the pathways through which they affect milk production traits. To our knowledge, no genes affecting mammogenesis have been directly linked to milk production. Recently, Raven et al. (2014) included traits of the mammary system in a GWAS study which identified five regions on four different chromosomes with significant effects; however, a clear description of the phenotype (the mammary system) was lacking.

**Table 1:** **Major genes involved in milk production**.

**Gene**	**Chr**.	Position (bp)^*^	**Trait**	**References**
*LEPR (leptin receptor)*	3	80,071,689–80,147,000	Milk yield Milk fat	Banos et al., 2008
*LEP (leptin)*	4	93,249,874–93,266,624	Milk yield Milk fat	Banos et al., 2008; Clempson et al., 2011
*IGF1 (insulin like growth factor 1)*	5	66,532,879–66,604,699	Milk yield Milk fat	Mullen et al., 2011; Waters et al., 2012
*ABCG2 (ATP-binding cassette, sub-family G, member 2)*	6	37,959,536–38,030,585	Milk protein Milk fat	Cohen-Zinder et al., 2005; Ron et al., 2006
*OPN (osteopontin)*	6	38,120,578–38,127,541	Milk yield Milk protein Milk fat	Leonard et al., 2005; Khatib et al., 2007
*PPARGC1A (peroxisome proliferator-activated receptor gamma, coactivator 1 alpha)*	6	44,854,113–44,960,533	Milk yield Milk fat	Khatib et al., 2007
*Casein-Cluster (CSN1S1, CSN2, CSN1S2, CSN3)*	6	87,141,556–87,392,750	Milk protein	Velmala et al., 1995; Kress et al., 2011
*DGAT1(diacylglycerol O-acyltransferase 1)*	14	1,795,425–1,804,838	Milk yield Milk fat	Winter et al., 2002; Strucken et al., 2010a
*BDNF (brain-derived neurotrophic factor)*	15	59,164,519–59,200,908	Milk fat	Zielke et al., 2011
*FTO (fat mass and obesity associated)*	18	22,118,201–22,541,540	Milk fat Milk protein	Zielke et al., 2013
*GHR (growth hormone receptor)*	20	31,890,736–32,064,200	Milk yield Milk protein Milk fat	Viitala et al., 2006; Banos et al., 2008; Rahmatalla et al., 2011
*PRLR (prolactin receptor)*	20	39,073,246–39,137,480	Milk yield Milk protein Milk fat	Bole-Feysot et al., 1998; Viitala et al., 2006; Lu et al., 2011b
*PRL (prolactin)*	23	35,105,135–35,113,750	Milk yield Milk protein Milk fat	Bole-Feysot et al., 1998
*AGPAT6 (1-acylglycerol-3-phosphate O-acyltransferase 6)*	27	36,212,352–36,228,987	Milk yield Milk fat	Bionaz and Loor, 2008a; He et al., 2011

* Btau_4.6.1.-Primary Assembly.

Only little is known in regards to time-dependent genetic effects causing a dynamic curve in dairy cattle but the next section summarizes the efforts and results made in this field.

## Dynamic genes in animal breeding systems

### Dynamic association studies

Whether a single marker for a candidate gene is used or thousands of indirect markers for a GWAS, finding associations between markers and a trait that displays dynamic expression over time can be difficult. The simplest solution may be to estimate associations over various time-points, i.e., treat each measurement as a separate phenotype. Automated milking systems could provide an accurate measurement of milk production for each day of lactation. However, this approach would mean that several hundred phenotypes would have to be analyzed. Further, whilst such measurements would provide daily milk yield, persistency cannot be estimated from a single time point. Ergo, daily measurements should not be treated as separate phenotypes. Therefore, appropriate phenotypic and genetic correlations have to be incorporated or repeated measurement analyses performed. Whilst daily measurements provide a highly accurate description of lactation performance, it might be computationally too time-consuming to be practically applied. Further, milking systems have still not penetrated the entire dairy sector and analyses solely relying on daily measurements would require additional methods to include animals with missing records. Most countries with national evaluation networks record milk production once a month. Assuming the lactation period of a cow lasts for 340 days, one record a month sums up to approximately 11 test-days. Because crucial changes such as peak yield occur roughly 6 weeks into the lactation cycle, one analysis every month could still give a fairly thorough picture of the lactation performance. However, analyzing 50k or 800k markers (the marker number of the most commonly used SNP-chip in dairy cattle at present) for thousands of animals would still take time.

Instead of using the measurements of the actual test-days, fewer parameters can be sufficient to describe an entire lactation. The profile of milk production, and its components, over the course of a single lactation has been described by various mathematical and biological functions (Pollott, 2004; Silvestre et al., 2009). Thus, using these mathematical lactation curve models provides a means of reducing the amount of time-points to a minimum of three curve parameters. These parameters describe the production curve through its properties such as slopes, apex (maximum), and level of production.

Such approaches are known as functional modeling in human genetics where it is mostly applied to map dynamic loci affecting disease traits using growth curves such as cubic splines (Hou et al., 2008; Li et al., 2009; Yang et al., 2009). In livestock research, a similar approach is known as the modeling of longitudinal or dynamic traits (Rodriguez-Zas et al., 2002; Suchocki and Szyda, 2011). In most livestock studies, the change in additive genetic variation over time was analyzed mainly for body weight and milk yield in dairy cattle, sheep, and goats (Lund et al., 2008; Roldan et al., 2008; Forni et al., 2009; Hadjipavlou and Bishop, 2009; Strucken et al., 2011). However, most of these studies used either no marker information or only a few markers on selected chromosomes to conduct their analyses.

The few results of time-dependent association studies in livestock reflect reported dynamic expressions of genes involved in milk production (Bionaz and Loor, 2008a; Verbyla and Verbyla, 2009) or add a time component to known but static effects of candidate genes such as the *DGAT1* gene or the region around the *casein* genes. The described effects of the *DGAT1* gene, with antagonistic impacts on milk yield and fat content, were shown to be detectable only after lactation day 40 (Strucken et al., 2011). This late effect points to a possible utilization of *DGAT1* in changing the persistency of milk production. Markers around the *casein* genes had strongest effects in early lactation (Strucken et al., 2012b), which is confirmed by the higher protein content in colostrum milk. Furthermore, investigations of the genes surrounding trait-associated markers showed that a substantial number of genes with stronger effects in early lactation are involved in immune response and not directly in milk production (Strucken et al., 2012b). Even though those genes have no direct effect on milk production, immune-related genes could influence the productivity of the animal by supporting udder health in a time of high activity (Wheeler et al., 2012; Chaneton et al., 2013) and through effects on food intake (Greer et al., 2008; Laurenson et al., 2011). This adds another group of genes that have to be considered when genetic influences on milk production traits are analyzed.

In general, the highest variation in associated loci were reported for early and late lactation, suggesting that those time periods provide the best opportunity for alteration through breeding schemes. This would also serve the idea of decreasing peak production through a slower increase in early lactation and increase the persistency of production in late lactation. Furthermore, by analyzing marker associations over time, we are more likely to find genetic markers with small effects over the whole lactation but strong effects at a specific time-point as they are not masked by major candidate genes such as *DGAT1*. Thus, time-dependent analyses could aid in detecting the missing genetic variance that explains the observed phenotypic variation.

Finally, differences in genetic effects were not only found for different lactation stages but also between lactations, especially between the first and later lactations (Strucken et al., 2012a). These differences between the first and later lactations are also observed in phenotypic production curves (Schmidt et al., 1988). Even though most cows are in puberty and have reached a sufficient weight and size to support a pregnancy at the age of first mating, first parity cows are still growing and the mammary gland undergoes the required changes to produce milk for the first time (Taylor et al., 2003, 2004). Therefore, this ongoing development during the first parity is most likely the reason for the lower performance compared to later lactations.

### Applications

In animal breeding, the ability of an animal to improve a trait in the next generation can be summarized using an estimated breeding value (EBV). The current standard for breeding value estimation is to include the animal's own performance records as well as the records of relatives, assuming that related individuals share a certain amount of genes with each other. In milk production, obviously a bull does not produce milk, and therefore its EBV is entirely dependent on milk production records of female relatives.

Because milk production is routinely recorded once a month in most countries, EBVs are based on these monthly test-day data. To account for the fluctuation of milk yield throughout a lactation, test-day models have been developed through the incorporation of appropriate lactation curve models (Misztal et al., 2000; Schaeffer et al., 2000; Swalve, 2000). Whilst some countries provide separate EBVs for persistency, most production EBVs are averaged over 305 days of lactation or even an average over several lactations. Therefore, the final selection decision is still based on a static value that makes it impossible to tell whether the animal had a high peak production or a good persistency. Though it should be easy for the national breeding evaluation centers to provide breeding values for certain time-periods (estimation equations implement lactation curve model), this would also increase the information output that needs to be handled and might even complicate the decision process about which animal should be used for mating.

One possible solution could be to include the shape of the production curve into the selection index and set a standard curve based on how much milk a cow can produce without inducing an energy deficiency under natural feeding conditions, and how much milk a cow should produce to be still profitable for the farmer. Based on such a standard curve, breeding values could be weighted according to their deviation from the standard curve resulting in a single EBV per animal.

A similar approach could be applied for genomically estimated breeding values (gEBVs) where the information of the genome-wide markers along with the production records of all relatives are included. gEBVs seem to be the way forward as they use the genetic constitution of the animal itself, and thus, what is actually inherited from generation to generation (Goddard and Hayes, 2007; Hayes et al., 2009; Hayes and Goddard, 2010). Nevertheless, gEBVs would require knowledge of either an optimal standard curve or the exact time-dependent genetic effects. Knowing the genetic effect of a marker enables us to simply genotype a selection candidate and sum up its genetic effects to calculate a gEBV, provided that the animals that were used to estimate the genetic effects are closely related to the selection candidate. If the actual causal mutation is known then family relations can be neglected.

Most of the reviewed studies on time-dependent genetic effects, applied a GWAS approach where the effects of each marker were estimated independently from all other markers. However, it is assumed that a quantitative trait such as milk production is shaped through the activity of many genes that might affect and even depend on each other. Thus, marker effects should not be estimated independently of all other markers in a study. A method which includes all markers at the same time has been termed Snp-BLUP, which is an extension of the original BLUP (best linear unbiased prediction) equation used to estimate the EBV of an animal (Goddard, 2009; Koivula et al., 2012).

Information obtained through dynamic GWAS or Snp-BLUP would make it possible to weight each marker according to its effects on the dynamic expression of the trait at different time-points, and thus provide a gEBV that includes the shape of the production curve. With the reducing cost of sequencing, causal genomic variants may be discovered and ultimately used in animal breeding to perform the most accurate selection possible.

## Concluding remarks

Milk production is a dynamic process and factors influencing this process occur as early as the fetal development. Whilst many physiological aspects of a dynamic milk production have been discovered, research on time-dependent genetic effects is still a wide open field. The animal breeding industry considers dynamic milk production by incorporating appropriate lactation curve models into their breeding value estimates to improve accuracy. Further, through an index of traits, breeders attempt to tackle the detrimental effects of a high milk production on other functional traits. However, if we assume that some of the negative issues arising from a high milk production can be overcome by altering the shape of the production curve, the impact of such an index on the actual dynamics of the lactation cycle are poorly understood. Since genetic and genomic tools are constantly developing with whole genome sequencing already being applied, our understanding of genes, their interactions and pathways will improve and direct causative mutations might be the target of future animal breeding programs. Understanding the time-dependent effects of genes and their variants is therefore an important field to study. Finally, whilst the dynamic of milk production is an obvious example, other time-dependent traits such as growth and weight gain, marbling, or onset of puberty could also benefit from a deeper understanding of the underlying dynamic of gene effects.

## Author contributions

EMS conceived of the topic and wrote the manuscript. YCSML and GAB advised and critically revised the manuscript.

### Conflict of interest statement

The authors declare that the research was conducted in the absence of any commercial or financial relationships that could be construed as a potential conflict of interest.

## Footnotes

^1^http://faostat.fao.org/site/569/DesktopDefault.aspx?PageID=569#ancor
